# Pitfalls in the Serological Evaluation of Maternal Cytomegalovirus Infection as a Potential Cause of Fetal and Neonatal Involvements: A Narrative Literature Review

**DOI:** 10.3390/jcm11175006

**Published:** 2022-08-26

**Authors:** Shigeo Iijima

**Affiliations:** Department of Regional Neonatal-Perinatal Medicine, Hamamatsu University School of Medicine, Hamamatsu 4313192, Japan; siijima@hama-med.ac.jp; Tel.: +81-53-435-2312

**Keywords:** antibody, cytomegalovirus infection, fetus, pregnancy, serological evaluation

## Abstract

Cytomegalovirus (CMV) is the most common cause of intrauterine infection and serological assays are the primary tools for assessing CMV infections during pregnancy. CMV-specific immunoglobulin M (IgM) antibodies have been used as a diagnostic marker for primary CMV infection in pregnant women, although CMV-IgM has been detected in non-primary CMV infections. IgG avidity testing may aid the distinguishing of primary from non-primary CMV infection; however, there is no standardized assay for detecting this difference. Moreover, when maternal serology shows positive CMV-IgG with negative CMV-IgM findings, vertical transmission probability following primary CMV infection is often excluded. However, symptomatic congenital CMV infections in the context of negative findings for maternal CMV-IgM have been reported recently. The absence of CMV-IgM is recognized in both primary and non-primary CMV infections. Furthermore, maternal non-primary CMV infections during pregnancy may yield a greater proportion of symptomatic congenital CMV infections than previously thought. If universal prenatal screening is performed, ultrasonography for abnormal fetal findings should be conducted regardless of CMV-IgM antibody status. If not universally screened, CMV antibody screening should be performed whenever routine fetal ultrasound reveals abnormal findings. For suspected fetal CMV infection, amniotic fluid or postnatal infant urine CMV-DNA testing is required.

## 1. Introduction

Cytomegalovirus (CMV) is the most common cause of congenital infection, affecting 0.5–5% of live births worldwide, and is a major cause of neurological and sensorineural impairments [1]. The majority of infants with congenital CMV infection are asymptomatic at birth and survive without sequelae, and only 10–15% of infected fetuses show signs of infection at birth [1]. However, the clinical manifestations (i.e., petechial rash, jaundice, hepatosplenomegaly, pneumonia, chorioretinitis, neurological abnormalities such as microcephaly and encephalitis, and/or fetal growth restriction with low birth weight) may be severe, leading to high levels of perinatal mortality and major neurological sequelae in most surviving infants. Moreover, 10–15% of asymptomatic neonates develop long-term sequelae such as progressive sensorineural hearing loss and intellectual disability [2]. Accurate measurement of the prevalence of this disease and its sequalae has eluded public health officials because not all neonates are tested for congenital CMV infection. However, early diagnosis and antiviral interventions can improve the prognoses of symptomatic infants [3]. Therefore, developing gold-standard prenatal detection methodologies to identify fetuses that are at risk of developing congenital CMV infection is of high priority. The virus transmission risk to the fetus is believed to be highest in pregnant women with primary CMV infections [4]. In addition, maternal serological screening is considered useful for identifying pregnant women with primary CMV infections, and maternal antibody tests detecting CMV-specific immunoglobulin (Ig) G and CMV-specific IgM antibodies are widely used [5]. However, routine prenatal screening for CMV via serology testing is currently not recommended by any public authority in any country for several reasons, including: (1) the unavailability of proven interventions, such as an effective vaccine for pregnant women who experience a primary CMV infection [5]; and (2) the concern that misinterpreted test results may have a strong influence on a woman’s decision to terminate the pregnancy. In the absence of routine serological CMV screening during pregnancy, most fetal CMV infections are diagnosed following the detection of ultrasonographic markers on routine scans. When fetal abnormalities suggestive of CMV infection are found on ultrasound, women are referred for further diagnostics, such as maternal serology testing. However, congenital CMV infections sometimes occur in infants born from pregnant women without detected CMV infections based on the results of antibody testing. In such cases, a diagnosis of a congenital CMV infection is achieved according to the infant’s symptomatology or fetal autopsy findings.

In this narrative review, the current state of knowledge on serological evaluations of maternal and fetal CMV infections is summarized. The most significant new findings concerning complex presentations with regard to maternal CMV serology are presented and the need for caution in interpretation is emphasized.

## 2. Literature Research Methods

A narrative approach was chosen to most effectively provide background information on serological evaluations of maternal and fetal CMV infections as well as comprehensively delineate the main topic of concern (i.e., challenges posed by complex presentations of maternal CMV serology). The literature review reported herein was conducted through systematic searches of established medical and scientific research databases, namely the PubMed, Google Scholar, ResearchGate, Cochran Library, Igaku Chuo Zasshi (ICHUSHI)-Web (i.e., Japanese medical literature) databases. In addition, a search using major Internet search engines (Google, Yahoo!) was conducted. Controlled and uncontrolled studies, meta-analyses, case series, case reports, and reports of animal models were included in the current review. Both English and Japanese-language papers were reviewed. A time window for the evaluated research publications was not specified. The literature search employed various keyword combinations: “congenital,” “primary,” “non-primary,” “cytomegalovirus or CMV,” “infection,” “pregnancy,” “fetus,” “neonate,” “serological,” “antibody,” “IgG,” “IgM,” “avidity,” “negative,” “positive,” “screening,” and/or “ultrasound.” Articles were screened by title and abstract, and subsequently, the full texts of the selected articles were examined. Articles that were irrelevant to the scope of this review were excluded. Additional literature was identified from the reference lists cited in the initially identified articles.

## 3. Transplacental Transmission of CMV Infection

CMV acquisition during pregnancy often occurs through contact with young children who may excrete CMV in saliva and urine, or through sexual contact, as CMV is present in semen [1]. It may be difficult to identify the onset of maternal CMV infection because symptoms suggestive of CMV rarely develop and CMV infections are thus rarely recognized based on symptomology alone. As maternal CMV infections can be active for months, transmission to the fetus likely occurs even weeks after the onset of maternal infection. CMV transmission from a mother to child can occur through three routes: intrauterine, intrapartum, and postnatal (i.e., during breastfeeding) [4]. Intrauterine transmission is the most important route and may be the result of transplacental virus transmission, followed by viral replication in multiple embryonic or fetal tissues [6]. There is little information on how the virus spreads; transmission may occur as a result of the hematogenous spread of maternal leucocytes across the placenta, or as a result of direct infection of the placental tissue and spreading to the amniotic cells that are then swallowed by the fetus and result in primary infection [7]. However, women with non-primary infection often lack evidence of viremia, suggesting vertical transmission by non-hematogenous routes.

**Figure 1:**
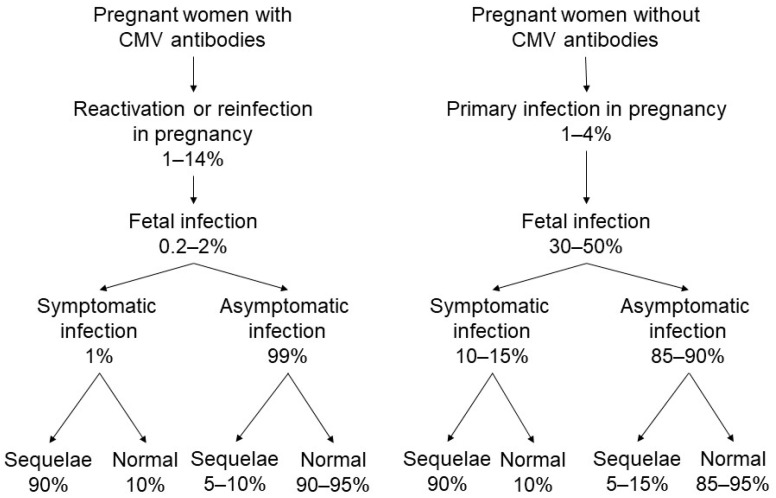
Cytomegalovirus (CMV) transmission from mother to fetus and potential disabilities in infants with congenital CMV infection.

Maternal CMV infection may be caused by a primary infection (i.e., first-hand experience with the virus) or a non-primary infection, as defined below [8]. Figure 1 shows a summary of the current state of knowledge regarding the epidemiology of CMV infections. For example, primary maternal infections occur in approximately 1–4% of seronegative pregnant women [8]. The risk of vertical transmission of CMV following a primary maternal infection has been estimated to be approximately 30–40% [9]. In addition, although maternal primary CMV infections occurring early in pregnancy are transmitted to only approximately 20% of exposed fetuses, early prenatal CMV infections are associated with severe symptomatic congenital CMV infections at birth and with severe and long-term sequelae [10,11]. Conversely, maternal infections in the third trimester have a maternal-fetal transmission rate of approximately 75%; however, symptomatic infections are uncommon, and are generally less severe when they do occur [12]. The human host never eliminates CMV, and persistent infection or reactivation of the latent virus may be involved in the epidemiology of this disease and its sequelae.

Non-primary maternal CMV infections occur when the mother has a history of CMV infection and is immune at the time of conception. This may be due to reactivation of the mother’s own latent strains or genotypes of CMV, or from a reinfection with a different (i.e., vertically transmitted) CMV strain or genotype [13]. Nearly all seropositive women reactivate CMV in breast milk with each subsequent pregnancy/lactation without viral systemic infection [14]. Moreover, a women could theoretically be infected with an antigenically identical strain in the absence of antigenic and/or genetic differences that would never be recognized as a different strain. Non-primary CMV infection in pregnancy occurs at a rate of approximately 1–14% [15]. The vertical transmission rate of CMV following a non-primary maternal infection is reported to be approximately 0.2–2% [15], and symptomatic congenital disease is diagnosed in less than 1% of known cases [8]. However, the biology of these infections, or why maternal immunity from previous infections is unable to prevent the occurrence of all congenital infections remain unclear.

Recently, it has been noted that congenital CMV infections may occur more often in infants of women who have preconceptional seropositivity [16]. In the United States, 75% of congenital CMV infection is attributable to maternal non-primary infections [17]. A registry-based cohort study in Finland reported that 54% of symptomatic congenital CMV infections were due to maternal non-primary infection [18], and Tanimura et al. reported in their prospective cohort study that 70% of infants with congenital CMV infections and 75% of infants with symptomatic congenital CMV infections were born from mothers with non-primary CMV infections [19]. Severe CMV-associated symptoms in fetuses and infants occurring as a result of maternal non-primary infection have similarly been reported within prior research [20]. Therefore, maternal non-primary infection is likely to contribute to more cases of congenital CMV disease than is currently documented [16].

## 4. Serological Confirmation of Maternal CMV Infection

The key purpose of maternal CMV screening is to establish laboratory confirmation of recent primary CMV infections, because >95% of pregnant women with primary CMV infections are asymptomatic and will thus rarely be diagnosed based on clinical symptomology alone [21]. Maternal serologic assays are the primary tools for identifying primary CMV infections during pregnancy. In commercial laboratories, serological testing modalities that detect CMV antibodies (IgM and IgG antibodies) are widely used. Additionally, evaluations of IgG avidity are considered confirmatory tests for identifying primary CMV infections. However, serological diagnosis of CMV infections is challenging, and the evaluation of CMV IgG, IgM, and IgG avidity is complex due to atypical response patterns and the diversity of test results that are obtained with various measurement kits.

### 4.1. Evaluation of CMV-IgG Antibodies

Following an initial CMV infection, the host begins to produce IgG antibodies to the virus within 1–2 weeks, and the production of CMV-specific IgG antibodies continues lifelong [22]. The most straightforward confirmation of primary CMV infection is determined based on findings of CMV-IgG seroconversion (i.e., conversion from negative to positive CMV-IgG antibody test findings). In a pregnant woman, the detection of CMV-IgG seroconversion is possible with paired of serum samples that can be used to identify infections based on pinpoint blood samples that are collected preconceptionally and during pregnancy [23]. However, such matched tests are rarely available during pregnancy. Moreover, in many countries, preconception serological screening for CMV is not recommended due to practical health-economics-related reasons or the uncertainty of serological testing [24]. Furthermore, seronegative women require periodic serological testing to detect seroconversion. A reassessment of CMV-IgG findings is usually performed early in the second trimester in order to detect cases of seroconversion. Reassessments are performed at least once during the third trimester (at 35–37 weeks of gestation) to identify neonates who are at risk of congenital CMV infection in cases of late seroconversion.

### 4.2. Evaluation of CMV-IgM Antibodies

**Figure 2:**
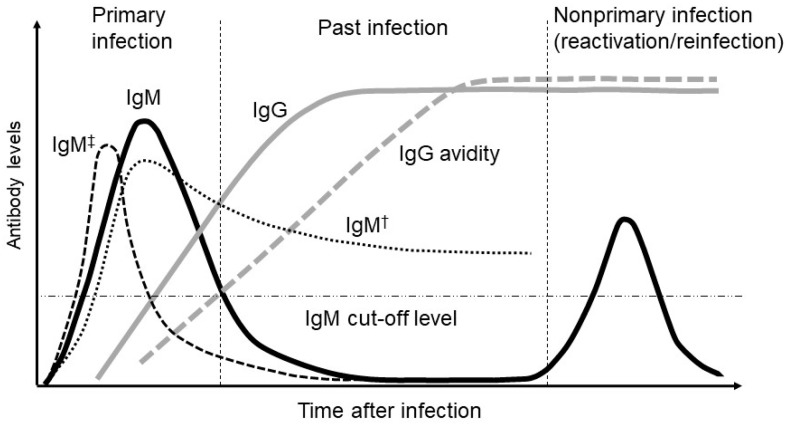
Relative changes in cytomegalovirus (CMV) IgM (immunoglobulin M), IgG (immunoglobulin G), and IgG avidity levels over time following a primary CMV infection. Another pattern of IgM presentation represents the long-term persistence of IgM (†) and the rapid clearance of IgM (‡) as an atypical IgM response.

CMV-IgM antibodies are generated following a primary CMV infection. More specifically, when examining CMV-IgM kinetics following a primary infection, peak levels are seen within the first 1–3 months, after which IgM titers decrease sharply within 2–3 months after the onset of infection and fall below the threshold of detection within 12 months [21] (Figure 2). Therefore, a diagnosis of primary CMV infection in pregnant women is most often based on a positive CMV-IgM antibody test, and the transient presence of specific IgM antibodies has long been used as a diagnostic marker for primary CMV infection. Sonoyama et al. reported that the probability of congenital CMV infection was appropriately 60% when pregnant women had positive CMV-IgM findings and fetal abnormalities on ultrasound [25].

However, the presence of CMV-IgM antibodies is not unique to primary CMV infections since assays for IgM antibodies lack specificity for primary infections. The CMV-IgM antibody has a high false-positive rate with regard to primary infections; <30% of pregnant women with positive IgM antibodies are determined to have a primary CMV infection [26]. Although the frequency is less than 10%, CMV-specific IgM production occurs during non-primary infections [21]. Moreover, CMV-IgM antibodies may be produced during reactivation or reinfection and may persist for more than a year following an acute primary infection [27,28]. Very rarely, a transient CMV-IgM response may occur with specific IgM antibodies persisting for a very short time and consequently going undetected even in the context of a recent primary infection [29]. Therefore, a positive CMV-IgM result alone is insufficient to accurately predict the risk of fetal infection. Moreover, false-positive IgM findings can result from aberrant and nonspecific cross-reactivity among several viruses (mostly herpes simplex virus, varicella-zoster virus, and Epstein–Barr virus infections) or due to interference from autoimmune antibodies such as rheumatoid factor [30,31].

In addition, the search for informative CMV-IgM tests has been hampered by the fact that the correlations of results obtained within different commercially available kits are poor and results are often contradictory [27,30]. Most commercially available serological kits use enzyme-linked immunosorbent assays (ELISAs). However, studies comparing the performances of CMV-IgM immune-enzymatic tests using native versus recombinant antigens demonstrated that recombinant antigens have lower sensitivities and specificities [32]. Moreover, Sarasini et al., reported that persistence of CMV-IgM positivity depended on the IgM method used. For example, 62% of women were still chemiluminescent immunoassay (CLIA) IgM-positive beyond 180 days after the onset of infection, whereas only 13% were ELISA IgM-positive [33]. Moreover, comparisons among ELISAs, microparticle enzyme immunoassays (MEIAs), and chemiluminescent microparticle immunoassays (CMIAs) have highlighted excellent agreement rates (more than 93%), but suboptimal correlations with regard to IgM detection (55–79%) [34]. In addition, a new assay for use on the Elecsys instrument (electrochemiluminescence immunoassay [ECLIA]) is more specific than the Architect assay (CMIA) for detecting CMV-IgM, and the Elecsys assay identified more negative results for persistent IgM than other assays [35]. These events can lead to false diagnoses of recent primary infections during pregnancy and can cause subsequent incorrect disease management, with potentially severe consequences. Therefore, positive CMV-IgM results should only be considered the starting point for a more detailed diagnostic evaluation in order to accurately determine whether the fetus is at risk for CMV infection.

With regard to the risk of fetal CMV infection, some investigators have reported that the influence of the dynamics of CMV-IgM antibody titers is currently unclear. Toriyabe et al. reported that high CMV-IgM titers in early pregnancy could predict the occurrence of fetal CMV infection [36]. Shimada et al. reported that CMV-IgM titers at CMV-IgG seroconversion were lower in women with (as compared to those without) fetal CMV infection [37].

### 4.3. Evaluation of CMV-IgG Avidity

IgG avidity describes the strength with which multivalent antibodies bind to multivalent antigens. IgG antibodies with low antigen avidity are present during the early weeks following a primary infection. Subsequently, IgG avidity gradually increases with time, reflecting the maturation of the immune response, and high IgG avidity then persists for many years [38,39] (Figure 1). CMV-IgG avidity assay is considered a primary tool to date the timing of CMV infection and represents a reliable commercial procedure for differentiating primary infections from non-primary infections in pregnant women. Most pregnant women with primary infections show low (<30%) IgG avidity during the first 3–4 months post-infection, followed by an intermediate range of avidity for 1–2 months and subsequent full IgG avidity maturation [40], reaching high levels (>65%) 5–6 months after the primary infection [26,39]. Therefore, the presence of reactive CMV IgM antibodies should be complemented by determining the maturity (avidity) of CMV IgG antibodies. Increasingly, tests for IgG avidity are used to distinguish primary infection from reactivation in IgM seropositive women. The presence of IgM antibodies in combination with low-avidity IgG antibodies provides strong evidence of a recent primary infection. In contrast, Ebina et al. reported that CMV IgG avidity with a cutoff value of less than 40% was clinically useful in the prediction of congenital CMV infections, especially prior to 28 weeks of gestation [41]. Additionally, Ebina et al. found that serum CMV IgG avidity increased more rapidly in pregnant women whose siblings were subsequently found to have congenital CMV infection than in women whose siblings had no congenital CMV infections [42].

However, CMV-IgG avidity test results can be misleading when used on sera that lack CMV-IgM antibodies and therefore have very low CMV-IgG antibody levels; a low IgG avidity result may be falsely presented in cases of preconception infections [43]. There are three important limitations with regard to CMV-IgG avidity assays: (1) the ranges of low- and high-avidity thresholds vary among the different commercially available kits [40]; (2) the timing of assay execution may critically affect negative predictive values (intermediate-to-high values obtained after 21 weeks of pregnancy cannot rule out a primary infection, while a high IgG avidity index detected in the first trimester properly identifies past infections) [21]; and (3) unusually long persistence (more than 18 weeks) of low IgG avidity may potentially lead to a misdiagnosis of a primary CMV infection, particularly when CMV-IgMs can also be detected [44]. Regarding (1), recent studies have reported good concordance between commercial assays for IgG avidity determination [45,46]. However, Sarasini et al. reported that approximately 6% of women whose sera were collected in the first 3 months after the onset of primary infection had high IgG avidity, whereas discordant results were observed with different avidity assays; these researchers suggested that further studies should be conducted in order to clarify this point [33]. Regarding (2), Kaneko et al. investigated serum samples of pregnant women with CMV-IgM positivity and demonstrated that low IgG avidity at ≤14 weeks of gestation was a good indicator of congenital infection [47].

Moreover, differing kinetics of IgG avidity maturation have recently been reported in pregnant women with primary infections [42]. A few CMV-IgM-negative patients have been reported to have low CMV-IgG avidity [39]. Thus, there is evidence that the duration and intensity of CMV viremia may directly affect the kinetics of the maturation of IgG avidity. In a prospective cohort study, Tanimura et al. demonstrated that maternal screening using CMV-IgG and avidity was inefficient with regard to the prediction of congenital CMV infection as this screening methodology overlooks more than half of pregnancies with congenital CMV infections arising from non-primary infections [19].

Commercial tests for CMV-IgG avidity are available in the United States [40]. However, these tests are not FDA-approved and require further standardization, and thus need to be used and interpreted with caution. In Japan, CMV-IgG avidity tests are not covered by the national health insurance.

## 5. Congenital CMV Infection in the Absence of Maternal CMV-IgM

In the absence of routine serological CMV screening during pregnancy, most fetal CMV infections are identified on routine ultrasound examination. Various sonographic markers are associated with fetal infections (Table 1) [29,48,49]. When fetal abnormalities suggestive of CMV infection are detected, women are referred for further diagnostics, including maternal serology testing and/or amniocentesis. When maternal serology shows positive CMV-IgG findings in the absence of CMV-IgM antibodies, the possibility of vertical transmission following a primary CMV infection is often ruled out simply because the result is suggestive of a previous CMV infection. Symptomatic congenital CMV infection is believed to be an extremely rare occurrence in patients with past infections [26]. However, studies on the implications of negative maternal IgM findings at the diagnosis of a fetal CMV infection are limited. Goncé et al. reported that maternal CMV-specific IgM antibodies were negative in half (56%) of infected pregnancies when ultrasound infection markers were detected [42]. In addition, our literature review identified 17 cases of confirmed congenital CMV infections in the absence of maternal CMV-IgM antibodies [50,51,52,53,54,55,56,57,58] (Table 2). The types of immunoassays that are used to measure CMV antibodies are known only in two cases (Noro’s case, EIA; Kawakami’s case, ELISA). In 16 (94%) of the 17 profiled cases, maternal CMV serology was only tested once when ultrasound abnormalities were observed; in 13 (76.4%) of the 17 cases, congenital CMV infection was diagnosed postpartum according to neonatal symptomology or fetal autopsy findings.

Maternal CMV-IgM evaluations have shown a low detection rate for non-primary CMV infections. Moreover, non-primary maternal infection has been reported to be associated with symptomatic congenital CMV infections [59,60]. Some investigators have reported that the manifestations, severity, and long-term prognoses with regard to this disease are similar when comparing primary and non-primary infections [20,61,62]. Maternal non-primary CMV infections during pregnancy can cause both asymptomatic and symptomatic congenital CMV. However, in a much larger percentage of neonates than previously thought, congenital CMV with severe manifestations has been reported [13,16,20,50,60,62,63,64,65,66]. Hadar et al. retrospectively investigated 107 neonates with congenital symptomatic CMV infections and demonstrated a statistically significantly low detection rate with regard to CMV-IgM antibodies when evaluating non-primary maternal infections occurring during pregnancy than that when evaluating primary infections (25% vs. 75.8%) [65]. In turn, some cases of congenital symptomatic CMV infection have been reported as arising from maternal primary infections with negative CMV-IgM findings. The IgM response may be very transient and undetected even in recent primary infections. Moreover, the absence of maternal CMV-specific IgM may be due to a negative response following primary maternal infection at a very early stage of pregnancy (the period of greatest fetal risk). In rare cases, specific IgM antibodies may be cleared rapidly, and a high IgG avidity could appear within 90 days after the onset of a primary infection [29]. If IgM-specific antibodies are absent and/or a high IgG avidity is detected in serum samples taken early after the onset of infection, a serological diagnosis of primary infection is very difficult to achieve. Sarasini et al. reported that both rapid clearance of specific IgM antibodies and fast IgG avidity maturation led to the misdiagnosis of 10.7% of recent CMV primary infections in the pregnant women enrolled in their investigation [33]. Moreover, Sarasini et al. described the early clearance of IgM antibodies during the first three months after the onset of a primary CMV infection in a population of pregnant women, in whom stringent diagnoses as well as the dating of primary infections were performed [33]. In this study, approximately 5% of women showed early clearance of CMV-specific IgM CLIA antibodies within three months after the onset of a primary infection. In addition, the absence of CMV-specific IgM ELISA antibodies was observed in approximately 15% of sera collected during the same time interval. Moreover, Kyriazopoulou et al. [67] investigated 32 women immunized against CMV with a normal pregnancy course and no suspicious ultrasound findings, and found 4 (12.5%) positive CMV results in amniotic fluid or fetal blood samples, even though none of the women had any serological evidence of maternal non-primary CMV infections.

## 6. Recommendations

The development and proper implementation of an effective vaccine holds promise for long-term interventions. However, despite ongoing research, no vaccine is currently available for preconceptional use to prevent congenital CMV infection [68]. It is unclear whether vaccines can prevent intrauterine infections. Moreover, possible reactivation of the attenuated vaccine virus during pregnancy raises additional safety questions. With regard to interventions for preventing fetal CMV infection, the use of CMV hyperimmune globulin did not significantly decrease the rate of vertical transmission in randomized clinical trials [29]. Recent randomized studies demonstrated that oral valaciclovir, an antiviral drug, effectively reduced the rate of vertical transmission of CMV following maternal primary infection early in pregnancy [69].

As most CMV infections are asymptomatic, the only way to detect a primary infection is to implement specific serological testing in pregnancy as early as possible. However, universal prenatal CMV screening has never been recommended by any country’s public health authority [24]. Therefore, structural or growth abnormalities seen on routine ultrasound examination are a frequent diagnostic starting point for determining maternal CMV infections. The sensitivity of this methodology is poor, and this diagnostic process correctly identifies no more than 20% of infected fetuses even in selected populations [49,70]. Moreover, abnormal fetal ultrasound findings predict symptomatic congenital CMV infections in only one-third of cases when fetal infection status is unknown [49]. However, a serological diagnosis of CMV infection is often difficult to perform in women whose serological status is unknown prior to pregnancy, and a high rate of neonates with congenital CMV infections are born to mothers with non-primary infections during pregnancy. Diagnosing a non-primary CMV infection during pregnancy is extremely challenging. For example, a non-primary infection cannot always be excluded in the case of negative CMV-IgM antibody findings. Moreover, virological or immunological markers for non-primary CMV infections have to date not been identified [1]. Table 3 shows the state of knowledge regarding the interpretation of CMV serology in pregnancy as well as the resulting implications for pregnant women, fetuses, and neonates.

Fetal infection can be diagnosed by CMV PCR evaluations of amniotic fluid, which are shown to have good sensitivity and a low associated risk of fetal loss (<1%) when carried out after 21 weeks of gestation and at least seven weeks after the onset of maternal infection (if known) [11,21,28]. Recently, as a less invasive test to replace amniocentesis, CMV enzyme-linked immunosorbent spot (ELISPOT) assay has been considered useful for predicting congenital CMV infection. This assesses maternal CMV-specific T cell-mediated immune responses based on interferon gamma produced by antigen-stimulated peripheral blood mononuclear cells [71]. However, this assay is non-standardized and not commonly available in daily practice.

Identification of neonates with congenital CMV infections through universal neonatal screening is likely the best methodology for detecting cases, including both symptomatic and asymptomatic cases at birth as well as cases born to mothers with either primary or non-primary CMV infections [72]. Early identification of these neonates would allow for providing proper anticipatory guidance with regard to detecting long-term health problems, such as hearing loss and neurodevelopmental disabilities, as well as with respect to providing appropriate antiviral treatments for neonates who may benefit from such therapy [73]. Currently, however, no universal neonatal screening programs have been developed or implemented.

Multiple diagnostic steps should be performed in order to diagnose maternal and fetal CMV infection. Ultrasonography for the detection of abnormal fetal findings should be conducted regardless of CMV-IgM antibody status, and CMV antibody screening should be performed whenever fetal ultrasound reveals abnormal findings. When the CMV-IgG antibody status is positive, CMV-DNA should be examined in amniotic fluid or postnatal infant urine regardless of CMV-IgM positivity.

## 7. Strength and Limitations

This narrative review was conducted because there are currently diverse data on congenital CMV infection and its prenatal diagnosis with regard to serological interpretations, and this area of interest needs to be mapped out for clinicians. Therefore, our review addressed a notable gap within the current literature.

However, this review has certain limitations. Its narrative design presents an inherent source of bias. Moreover, only one researcher (the single author of this manuscript) determined the study selection. These issues may have resulted in selection bias. However, the author believes that the search was comprehensive with regard to the current state of the literature regarding congenital CMV infection and its prenatal serological evaluation. Furthermore, to reduce the impact of publication bias, this review included gray literature, books, and monographs in addition to peer-reviewed journals, as these sources provide practical experiences and important information on congenital CMV infection.

Second, the majority of studies reporting on congenital CMV infection in the absence of maternal CMV-IgM were published in Japanese-language journals (i.e., were written in Japanese). In Japan, universal screening for CMV primary infections among pregnant women is not recommended, though random screening has been conducted in some institutions. Of note, the prevalence of maternal CMV seropositivity and of identified patients with congenital CMV infections in Japan is similar to the corresponding figures reported in other countries [74,75]. Therefore, the author believes that congenital CMV infection in the absence of maternal CMV-IgM antibodies is not a frequent occurrence in Japan. Nevertheless, comprehensive mapping of the various studies reported to date can provide clinicians with valuable information on the advantages and disadvantages of the evaluated diagnostic methodology. This is a substantial strength of this work.

## 8. Conclusions

Appropriate serological screening of pregnant women with regard to CMV infection has remained a key component and focus of studies on both maternal and fetal CMV infections. However, the interpretation of negative or positive CMV-IgM findings in pregnant women is highly complex, and some cases of congenital symptomatic CMV infection arising from primary maternal infection with positive CMV-IgG and negative CMV-IgM findings have been reported. The development of accurate, standardized, and widely available assays for CMV-IgM antibodies and IgG avidity could change the risk–benefit ratio with respect to maternal screening for CMV. At this time, multiple diagnostic steps, including serological, ultrasound, and PCR examinations, should be performed in order to diagnose maternal, fetal, and neonatal CMV infections accurately and with consistency. Our findings thereby guide future research directions while also directly informing medical guidelines and effective clinical decision-making.

## Figures and Tables

**Table 1:** Ultrasound fetal abnormalities suggestive of congenital cytomegalovirus infection.

Placental or Amniotic Fluid Abnormalities	Cranial Abnormalities	Extracranial Abnormalities
Placentomegaly Placental calcifications Oligohydramnios Polyhydramnios	Ventriculomegaly * Microcephaly * Intracerebral calcifications * Increased periventricular echogenicity Calcifications of the lenticulostriate vessels Intraventricular synechiae Periventricular pseudocysts Subependymal cysts Choroid plexus cysts Increased cisterna magna Polencephaly Lissencephaly Callosal dysgenesis Increased cisterna magna Vermian hypoplasia Cerebellar hemorrhage Cerebellar calcifications Cerebellar cysts	Intrauterine growth restriction * Ascites * Hepatosplenomegaly * Hyperechogenic bowel Intrahepatic calcifications Pleural effusion Pericardial effusion Subcutaneous edema Hydrops fetalis

* High prevalence.

**Table 2:** Cases of symptomatic congenital cytomegalovirus (CMV) infection in the absence of maternal CMV-IgM (immunoglobulin M) antibodies.

Case	Maternal Age	GA at First Presentation (Weeks)	Fetal Abnormalities	Maternal CMV Serology				Fetal CMV Testing	GA at Birth (Weeks)	BW (g)	Sex	Neonatal CMV Testing			Neonatal Abnormalities	Neonatal Outcomes
				GA at First Testing (Weeks)	IgG	IgM	IgG Avidity	Amniotic Fluid DNA				IgG	IgM	DNA		
Henrich, 2002 [51]	40	20	Fetal ascites; fetal echogenic bowels; fetal enlarged heart; ventricular dilatation; intracerebral calcification	20	P	N	NA	P	33	1820	M	P	P	NA	Massive abdominal ascites; hepatosplenomegaly; extramedullary blood synthesis	Death
Mizuno, 2012 [52]	29	19	Oligohydramnios; fetal growth restriction; microcephaly	22	P	N	NA	NA	28	894	UK	NA	P	P (U)	Microcephaly; hepatosplenomegaly; generalized petechiae; DIC	NDI
	26	22	Fetal ascites; microcephaly; ventricular dilatation	22	P	N	NA	NA	39	2936	UK	NA	P	NA	Microcephaly; ventricular dilatation; polymicrogyria	NDI
	28	34	Microcephaly	36	P	N	NA	NA	36	2786	UK	NA	P	NA	Mild respiratory distress	Normal
Okumura, 2013 [53]	UK	31	Ventriculomegaly	33	P	N	NA	NA	40	2836	UK	NA	NA	P (B)	Ventriculomegaly; subcortical white matter abnormality; cystic lesions in temporal regions	NDI
Noro, 2016 [54]	32	20	Fetal ascites	20	P	N	NA	NA	32	2588	F	P	P	P (U)	Ascites; pulmonary hypoplasia; anasarca; encephalodysplasia; thrombocytopenia	Death
Kawakami, 2016 [55]	29	24	Fetal ascites; fetal echogenic bowels; fetal growth restriction; fetal anemia	24	P	N	NA	P	26	610	F	NA	NA	NA	CMV placentitis	Death ^†^
Gunkel, 2017 [50]	UK	20	Fetal echogenic bowels; lenticulostriate vasculopathy	32	P	N	High	NA	40	3460	F	NA	NA	N (U)	Hepatosplenomegaly; lenticulostriat1e vasculopathy; white matter calcifications; germinolytic cysts; widespread petechiae; thrombocytopenia,	NDI
	UK	30	Ventriculomegaly	32	P	N	High	NA	38	2750	F	NA	NA	P (U)	Ventriculomegaly; lenticulostriate vasculopathy; germinolytic cysts; thrombocytopenia	Normal
	UK	21	Oligohydramnios; hydrops fetalis; fetal enlarged heart; fetal echogenic bowels; thickened nuchal fold	21	P	N	High	P	23	573	M	NA	NA	NA	CMV positive immunohistochemical staining in the pancreas, spine, liver, lung, kidneys, and placenta; CMV inclusion bodies in the brain	Death ^‡^
	UK	21	Microcephaly; cerebellar hypoplasia	21	P	N	High	NA	22	595	F	NA	NA	NA	Microcephaly; CMV inclusion bodies in the kidneys and brain	Death ^‡^
	UK	22	Oligohydramnios; fetal echogenic bowels	22	P	N	High	NA	37	2890	F	NA	NA	P (U)	Hepatosplenomegaly; ventriculomegaly; lenticulostriate vasculopathy; polymicrogyria; intracranial hemorrhage; widespread petechiae; thrombocytopenia; CMV chorioretinitis	Death
Toyoda, 2017 [56]	28	27	Ventriculomegaly	29	P	N	NA	NA	37	1891	M	P	P	P (U)	Ventriculomegaly; periventricular calcification; CMV chorioretinitis	UK
Tachi, 2018 [57]	UK	31	Polyhydramnios; fetal ascites; hydrops fetalis; ventriculomegaly; esophageal atresia	31	P	N	NA	NA	33	1602	UK	NA	NA	P (U)	Ventriculomegaly; esophageal atresia	UK
	UK	26	Fetal ascites; bowel dilatation; ventriculomegaly	26	P	N	NA	P	32	2224	UK	NA	NA	P (U)	Meconium peritonitis; thrombocytopenia	UK
	UK	34	Ventriculomegaly	34	P	N	NA	NA	37	2654	UK	NA	NA	P (U)	CMV retinitis; hearing impairment	UK
Chan, 2020 [58]	30	26	Fetal ascites	26	P	N	NA	NA	36	3020	F	NA	NA	NA	Ascites; meconium peritonitis; intestinal malrotation, pulmonary hypoplasia; CMV immunoreactivity in lungs, liver, and kidneys	Death

†, intrauterine fetal death; ‡, termination. IgG, immunoglobulin G; IgM, immunoglobulin M; GA, gestational age; BW, birth weight; P, positive; N, negative; NA, not applicable; M, male; F, female; NDI, neurodevelopmental impairment; UK, unknown; U, urine; B, blood; DIC, disseminated intravascular coagulation.

**Table 3:** Interpretation of cytomegalovirus (CMV) serology in pregnancy.

Indications for CMV Screening	CMV Antibodies	IgG Avidity	Interpretation	Implications for the Pregnant Woman	Implications for the Fetus and Neonate
Universal prenatal screening Maternal flu-like illness Structural or growth abnormalities of fetus on prenatal ultrasound examination	IgG− IgM−	NA	Uninfected or early infection	Hygiene and behavior measures Consider repeat serological testing Seroconversion: primary infection No seroconversion: serological screening at 35–37 weeks of gestation	Not a past infection: Fetal diagnosis by ultrasonographic evaluation and a CMV-DNA assay of the amniotic fluid (if possible) Neonatal diagnosis by a CMV-DNA assay of the urine
	IgG− IgM+	NA	Very recent infection May be false positive due to other viral infections	Repeat serological testing in two weeks Perform IgG avidity if IgG positive	
	IgG+ IgM−	NA	Past infection or non-primary infection	CMV IgG and IgM at every trimester of pregnancy Significant rise (at least two-fold) in serial IgG titers: absence of past infection IgG avidity testing if it is clinically warranted	
	IgG+ IgM+	High	Past infection or non-primary infection May be primary infection	CMV IgG and IgM at every trimester of pregnancy Significant rise (at least two-fold) in serial IgG titers: absence of past infection	
	IgG+ IgM+	Low	Recent primary infection May be non-primary infection	CMV IgG and IgM at every trimester of pregnancy	

NA, not applicable; IgG, immunoglobulin G; IgM, immunoglobulin M.

## Data Availability

Not applicable.
